# Laparoscopic Fertility-Sparing Surgery for Early Ovarian Malignancies

**DOI:** 10.3390/cancers15205099

**Published:** 2023-10-22

**Authors:** Julia S. M. Zimmermann, Pauline Ramisch, Marc P. Radosa, Christoph G. Radosa, Askin C. Kaya, Sara Y. Brucker, Florin A. Taran, Uwe A. Ulrich, Andreas Hackethal, Martin Deeken, Marc Sütterlin, Benjamin Tuschy, Erich-Franz Solomayer, Julia C. Radosa

**Affiliations:** 1 Department of Gynecology, Obstetrics and Reproductive Medicine, Saarland University Hospital, D-66421 Homburg, Germany; julia.zimmermann@uks.eu (J.S.M.Z.);; 2 Department of Gynecology and Obstetrics, Klinikum Bremen Nord, D-28755 Bremen, Germany; 3 Institute and Policlinic of Diagnostic and Interventional Radiology, Medical University, TU Dresden, D-01307 Dresden, Germany; 4 Department of Gynecology, Tübingen University Hospital, D-72076 Tübingen, Germany; 5 Department of Gynecology, University Medical Center Freiburg, D-79106 Freiburg, Germany; 6 Department of Gynecology, Martin Luther Hospital, Johannesstift Diakonie, D-14193 Berlin, Germany; 7 Frauenklinik an der Elbe, D-20457 Hamburg, Germany; 8 Department of Gynecology, D-66346 Püttlingen, Germany; 9 Department of Gynecology and Obstetrics, University Medical Center Mannheim, Heidelberg University, D-68167 Mannheim, Germany

**Keywords:** fertility-sparing surgery, early ovarian cancer, laparoscopy, oncologic safety, reproductive outcomes

## Abstract

**Simple Summary:**

The surgical treatment of ovarian cancer traditionally includes bilateral adnexectomy and hysterectomy and thus terminates patients’ fertility. The demand for fertility-sparing surgical treatment options for gynecological cancers has increased in the last decade due to socioeconomic changes such as increased maternal age at first pregnancy, increased occurrence of malignancies in younger patients, and technical progress in surgery that enables such options. Randomized controlled trials examining the fertility-sparing treatment of ovarian cancer are lacking, but retrospective studies, including primarily patients treated with open surgery, have yielded comparable survival data with acceptable fertility outcomes and provide a rationale for the offering of fertility-sparing options to selected patients with early ovarian cancer. The aim of this study was to accumulate additional evidence on oncological safety and fertility outcomes in patients with early-stage ovarian cancer treated with laparoscopic fertility-sparing surgery.

**Abstract:**

The demand for fertility-sparing surgery (FSS) has increased in the last decade due to increased maternal age, increased incidence of ovarian malignancies in younger patients, and technical advances in surgery. Data on oncological safety and fertility outcomes of patients with ovarian cancer after laparoscopic FSS are sparse, but some retrospective studies have shown that open FSS may be offered to selected patients. We assessed the role of minimally invasive FSS in comparison with radical surgery (RS) in terms of oncological safety and reproductive outcomes after FSS in this multicenter study. Eighty patients with FIGO stage I/II ovarian cancer treated with laparoscopic FSS or RS between 01/2000 and 10/2018 at the participating centers (comprehensive gynecological cancer centers with minimally invasive surgical expertise) were included in this retrospective analysis of prospectively kept data. Case–control (*n* = 40 each) matching according to the FIGO stage was performed. Progression-free survival [150 (3–150) and 150 (5–150) months; *p* = 0.61] and overall survival [36 (3–150) and 50 (1–275) months; *p* = 0.65] did not differ between the FSS and RS groups. Eight (25.8%) women became pregnant after FSS, resulting in seven (22.5%) deliveries; three (37.5%) patients conceived after in vitro fertilization, and five (62.5%) conceived spontaneously. Laparoscopic FSS seems to be applicable and oncologically safe for patients with early-stage ovarian cancer, with adequate fertility outcomes.

## 1. Introduction

With 220,000 new cases diagnosed annually, ovarian cancer is among the most frequently occurring cancers in women and the second leading cause of death from gynecological malignancies worldwide [1,2]. Despite the optimization of chemotherapeutic regimes and the development of new therapies, surgery, including hysterectomy and bilateral oophorectomy, has been the standard ovarian cancer treatment for decades [3]. Although ovarian cancer is most frequently diagnosed in postmenopausal women, its incidence is increasing in patients under 40 years of age, who comprise 3–17% of all patients with epithelial ovarian cancer [4,5,6,7]. A constant increase in maternal age at first birth has been observed in developed countries over the past 20 years [8], accompanied by an increasing number of patients affected by ovarian cancer before the termination of their future childbearing desire [9]. As the standard surgical treatment of ovarian cancer results in infertility, the role of fertility-sparing surgery (FSS) for young patients needs to be assessed [10]. FSS is defined by the preservation of the uterus and at least one ovary and includes cystectomy, unilateral oophorectomy, and unilateral adnexectomy [11]. Based on recent research findings, individualized cancer therapies with fertility-sparing options have become possible for selected patients, but current data on oncological and fertility outcomes and the safety of these procedures are derived from retrospective series, primarily of patients with International Federation of Gynecology and Obstetrics (FIGO) stage I ovarian cancer treated with open surgery, with insufficient follow-up periods [3]. Based on the National Comprehensive Cancer Network guidelines, FSS can be offered to patients with stage I epithelial ovarian cancer; the role of minimally invasive surgery in the treatment of ovarian cancer, however, remains controversial, and data on a laparoscopic approach are lacking [7,12]. According to the European Society of Gynecological Oncology, FSS is indicated only following adequate surgical staging in patients younger than 40 years, when it is performed by an expert surgeon in a tertiary center, the patient is compliant with a strict follow-up protocol, and a designated gynecological pathologist performs the evaluation [13]. Sufficient pretreatment counseling with the discussion of FSS options is mandatory for patients of reproductive age [14,15,16]. Points of interest include the potential impacts of concomitant oncological therapies and the impact of FSS on the patient’s fertility; more research on outcomes following such surgery is needed to guarantee the adequacy of pretreatment counseling [14]. Thus, we compared the oncological safety and reproductive outcomes of minimally invasive FSS and radical surgery (RS) in a multicentric study, including patients with early ovarian cancer.

## 2. Materials and Methods

### 2.1. Study Design and Patient Selection

All patients presenting with newly diagnosed early (FIGO stages I and II) invasive ovarian cancer between January 2000 and October 2018 at the participating centers were screened retrospectively for study inclusion by review of prospectively kept service databases. The participating sites were comprehensive cancer centers in Germany with extensive experience in minimally invasive surgery: the Department of Gynecology, Obstetrics, and Reproductive Medicine at Saarland University Hospital; the Department of Gynecology at Tübingen University Hospital; the Department of Gynecology at Martin Luther Hospital, Berlin; the Day-care Hospital at Altonaer Strasse, Hamburg; and the Department of Gynecology and Obstetrics at Mannheim University Hospital. The inclusion criteria were ovarian cancer treatment with laparoscopic FSS or RS (control) at one of the participating sites and the availability of medical charts of complete data on the study variables. To balance the patient and tumor characteristics and potential confounding factors between the FSS and RS groups, case–control matching was performed according to the FIGO stage [3]. Patients with non-invasive (borderline) or non-epithelial ovarian tumors, those with advanced (FIGO stages III and IV) cancer as indicated on final pathology reports, and those whose chart information was incomplete were excluded. The study was approved by the Saarland Institutional Review Board (no. 155/17) and registered in the German trial registry (DRKS00013084).

### 2.2. Data Collection

The following clinical and pathological data were collected by chart review: patient age, FIGO ovarian tumor stage, histological subtype, surgical technique (FSS or RS), number of intraoperative complications (defined as bleeding, organ injury, resuscitation and skin emphysema), postoperative complications (defined as all deviations from the normal postoperative course occurring during a six-week period after surgery) graded according to Clavien Dindo, postoperative length of hospital stay, additional oncological treatment (i.e., adjuvant chemotherapy), outcomes [progression-free survival (PFS) and overall survival (OS)], and follow-up data [3,17]. For the FSS cohort, data on the time to pregnancy after oncological surgery, postsurgical reproductive outcomes (e.g., numbers of full-term pregnancies and miscarriages), in vitro fertilization (IVF) and spontaneous conception, gestational age at time of delivery, delivery mode, and the performance of completion surgery (radical resection of the uterus and remaining ovary) after family completion were also collected.

### 2.3. Treatment Procedures

FSS and RS were performed according to international guidelines for the stage-adjusted treatment of ovarian cancer [13]. All patients undergoing FSS underwent detailed preoperative counseling, including the discussion of all potential benefits and risks of the less-radical surgery. FSS consisted of cystectomy, oophorectomy, or oophorectomy and contralateral cystectomy, and RS consisted of bilateral oophorectomy and hysterectomy. Complete staging (peritoneal cavity exploration, multiple peritoneal biopsies, pelvic washings, infracolic omentectomy, and pelvic and para-aortic lymphadenectomy) was performed in all patients according to the national guidelines for ovarian malignancies [18]. Board-certified gyneco-oncologists with extensive experience in minimally invasive surgery performed all procedures with patients under general anesthesia using standardized techniques [16]. All patients with stage IA/B (high-grade) or stage IC or more advanced disease received adjuvant platinum-based chemotherapeutic regimens following international guidelines [16].

### 2.4. Statistical Analysis

The data were managed and analyzed using SPSS Statistics (v. 19; IBM, Chicago, IL, USA). The normality of data distribution was assessed using the Kolmogorov–Smirnov test, which showed a non-Gaussian distribution. The FIGO stage was selected as a conditioning variable, as it was the only variable relevant to disease-free survival and OS in a multivariate binary logistic regression analysis performed prior to target population selection, but not balanced in the FSS or RS setting. Qualitative and quantitative data are presented as absolute and relative frequencies and medians with ranges, respectively. The data were analyzed using the Mann–Whitney *U* test with post-hoc Bonferroni correction. Categorical variables were compared between groups using Pearson’s chi-squared test. The Kaplan–Meier method was used for the univariate analysis of PFS and OS (months from initial surgery to recurrence and death, respectively). The survival curves between the FSS and RS groups were compared using the log-rank test. Univariate binary logistic regression analyses of clinically relevant factors associated with PFS and OS were conducted. Two-tailed *p* values < 0.05 were considered to be significant.

## 3. Results

### 3.1. Patient and Tumor Characteristics

Of 103 patients with early ovarian cancer who underwent laparoscopic surgery at the participating centers, 23 were excluded due to incomplete chart data. The final sample comprised 40 women who underwent FSS and 40 matched women who underwent RS.

The median ages in the RS and FSS groups were 56 (range, 29–81) and 28 (range, 15–54) years, respectively (*p* ≤ 0.01). In each cohort, 23 (57%) patients had FIGO stage IA, no (0%) patient had stage IB, 10 (25%) patients had stage ICI, 2 (5%) patients had stage ICII, 3 (8%) patients had stage ICIII, and 2 (5%) patients had FIGO stage II disease. Twenty-two (55%) women who underwent RS and 13 (32%) women who underwent FSS had serous ovarian cancer (*p* ≤ 0.01). In the FSS cohort, 21 (52%) patients had low-grade (G1) tumors, 11 (28%) had G2 tumors, and 8 (20%) patients had high-grade (G3) tumors. The corresponding numbers in the RS cohort were 11 (28%), 16 (40%), and 13 (32%), respectively (*p* = 0.07). Twelve (30%) patients in the FSS group and 17 (43%) patients in the RS group received adjuvant platinum-based chemotherapy consisting of carboplatin (area under the curve five every three weeks intravenous, Fresenius Kabi Deutschland GmbH, Bad Homburg, Germany), alone or in combination with paclitaxel (175 mg/m^2^ every three weeks intravenous, Fresenius Kabi Deutschland GmbH, Bad Homburg, Germany; *p* = 0.25). In one case (2%), an intraoperative complication in the form of an iatrogenic bowel lesion occurred in the RS group. No intraoperative complication occurred in the FSS group (*p* = 0.31). Postoperative complications occurred in five (12%) and three (7%) patients in the RS and FSS groups, respectively (*p* = 0.46). The median duration of postoperative hospital stay was 9.5 (0–27) days in the RS group and 4 (0–12) days in the FSS group (*p* = 0.04). Histopathological characteristics and details on surgical complications are presented in Table 1.

### 3.2. Oncological Outcomes

**Figure 1:**
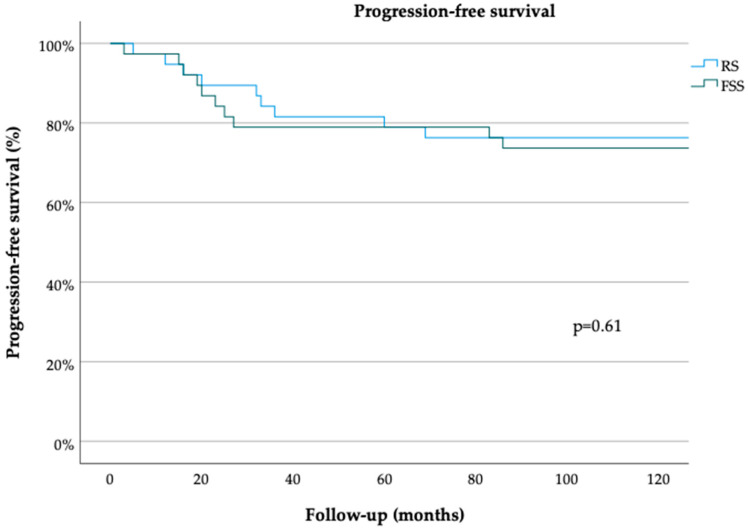
Progression-free survival for minimally invasively treated patients with early ovarian cancer. Radical surgery (RS), fertility-sparing surgery (FSS) (log-rank test).

**Figure 2:**
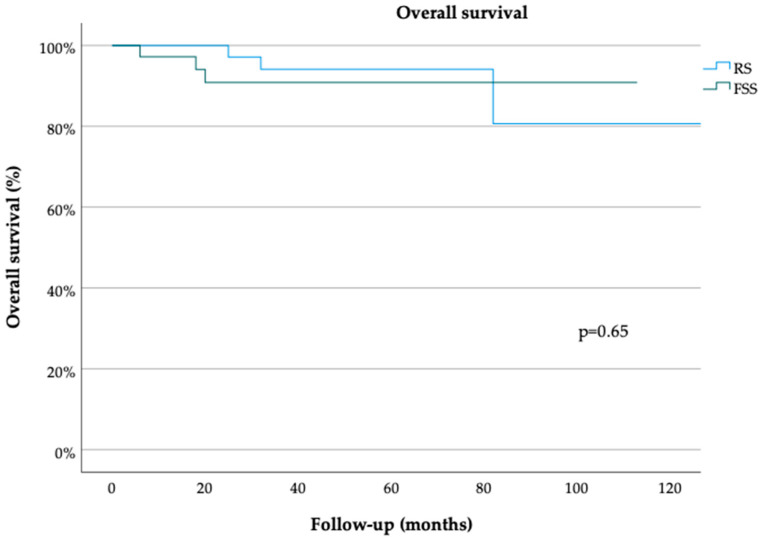
Overall survival for minimally invasively treated patients with early ovarian cancer. Radical surgery (RS), fertility-sparing surgery (FSS) (log-rank test).

The median follow-up durations in the FSS and RS groups were 36 (range, 3–150) and 54 (range, 1–275) months, respectively (*p* = 0.69 Table 1). The median PFS durations in the FSS and RS groups were 150 (range, 5–150) and 150 (range, 3–150) months, respectively (*p* = 0.61) (Figure 1). The 3- and 5-year PFS rates were 80% [standard error (SE) = 0.06] and 68% (SE = 0.09), respectively, in the FSS group and 85% (SE 0.06) and 72% (SE 0.08), respectively, in the RS group (both *p* = 0.78) (Table 1). The median OS durations in the FSS and RS groups were 35 (range, 3–150) and 50 (range, 1–275) months, respectively (*p* = 0.65) (Figure 2). The 3- and 5-year OS rates were 90% (SE 0.06) in the RS group (*p* = 0.61) (Table 1). On univariate analysis, PFS was not associated with the patient age [odds ratio (OR 0.99, 95% confidence interval (CI) 0.97–1.03, *p* = 0.81], surgical approach (FSS vs. RS; OR 0.76, 95% CI 0.27–2.12, *p* = 0.60), FIGO stage (OR 6.11, 95% CI 0.52–71.44, *p* = 0.15), tumor grade (OR 0.84, 95% CI 0.44–1.61, *p* = 0.59), or histological subtype (serous vs. endometrioid; OR 1.05, 95% CI 0.26–4.24, *p* = 0.95) (Table 2). OS also was not associated with the patient age (OR 1.01, 95% CI 0.97–1.06, *p* = 0.57), surgical approach (OR 1.06, 95% CI 0.20–5.61, *p* = 0.95), FIGO stage (OR 0, 95% CI 0–1, *p* = 0.99), tumor grade (OR 2.17, 95% CI 0.73–6.42, *p* = 0.16) or histological subtype OR 0, 95% CI 0–1, *p* = 0.99) (Table 3). Multivariate analysis was not performed due to the lack of association of these clinicopathological confounders in the univariate analyses.

### 3.3. Pregnancy Outcomes

Follow-up fertility information was obtained for 31 (77.5%) patients who underwent FSS. The median time from oncological surgery to pregnancy was 12 (range, 6–37) months. Eight (26%) women had at least one pregnancy after FSS; three (38%) conceived with IVF, and five (63%) conceived spontaneously. One (3%) woman did not conceive after IVF, and two (6%) women cryopreserved oocysts. Seven (23%) women were pregnant once, and one (3%) woman was pregnant twice, resulting in seven (23%) full-term pregnancies and two (6%) miscarriages. The median gestational age at the time of delivery was 38 (range, 33–40) weeks. Five (72%) women delivered spontaneously, and two (28%) women delivered by cesarean section. Three (8%) women underwent completion surgery after delivering, and four (10%) women had completion surgery without realizing their childbearing desires. The live birth rate in this cohort was 225 per 1000, and the baby take-home rate was 75% (Table 4).

## 4. Discussion

In this multicentric case–control study that included 80 patients who underwent laparoscopic FSS or RS for early-stage ovarian cancer, we observed no significant difference regarding the surgical technique performed (FSS vs. RS) and PFS or OS. In the FSS cohort, the baby take-home rate was 75%.

Our results regarding the oncological safety of laparoscopic FSS for early-FIGO-stage ovarian cancer are consistent with recent findings for open FSS. In a retrospective single-center study, Wright et al. [19] demonstrated that ovarian conservation (*n* = 432) had no effect on survival relative to bilateral oophorectomy (*n* = 754) for FIGO stages IA and IC ovarian cancer in women aged < 50 years. They thus characterized ovarian conservation as safe in this patient group and identified the tumor grade and stage as predictive of survival [19]. These results are consistent with our findings, but Wright and colleagues did not consider FIGO stages other than IA and IC. Similarly, Fruscio et al. retrospectively evaluated the outcomes of patients with epithelial ovarian cancer who underwent FSS (*n* = 240) or RS (*n* = 789; 91.5% of all patients underwent open surgery) over a median follow-up period of nine years. Their multivariate analysis revealed that the tumor grade, but not the surgical approach, negatively affected prognosis [20]. These findings suggest that FSS is feasible for patients with stage I epithelial ovarian cancer, but the authors did not match patients undergoing FSS and RS based on tumor biology or stage and did not statistically balance the cohorts regarding those characteristics, leaving space for potential selection bias [20]. Crafton et al. analyzed national data for the period 1992–2014 on the oncological outcomes of 9017 women aged 15–44 years with FIGO stages I–IV epithelial ovarian cancer who underwent FSS or RS in the United States and found that the use of FSS to treat early ovarian cancer did not affect survival in subgroups defined by stage and grade, in line with our observations. They noted that the OS rate was lower among women with stages II–IV high-grade disease who underwent FSS relative to those who underwent RS [21]. We observed no difference associated with FIGO stage II in this study, but as our cohort included only two patients with stage II disease and no patient with stage III or IV disease, we cannot comment on this finding in a statistically reliable manner.

Although the mostly retrospective findings on the use of laparoscopic FSS for the treatment of early ovarian cancer hint at its oncological safety, the laparoscopic treatment of ovarian cancer remains controversial as its risks and benefits relative to those of conventional open laparotomy remain unclear. Some authors have pointed out that comprehensive surgical staging via laparoscopy may be technically difficult [22]. In addition, laparoscopy has been associated with a higher rate of intraoperative cyst rupture, resulting in upstaging [23]. Other points of concern may include the occurrence of port-site metastasis, although this risk is considered to be low for early-stage ovarian cancer, and the controversial potential impacts of pneumoperitoneum on intra-abdominal tumor cell carryover and growth factor production [24,25]. Several retrospective studies have shown that the application of laparoscopy in the treatment of early ovarian cancer is safe and feasible. Ghezzi et al. compared the oncological outcomes of 34 patients with early-stage ovarian cancer treated with laparoscopy or laparotomy and found laparoscopy to be safe and technically feasible [26]. The largest published study on this topic is a multicenter retrospective series of 300 patients with early ovarian cancer treated laparoscopically, which showed that the recurrence, PFS, and OS rates were comparable to those reported in the literature for open surgical treatment [27]. Despite the lack of randomized controlled trials addressing this topic, laparoscopic surgery for early ovarian cancer seems to be feasible and oncologically safe, and this procedure has been implemented gradually in the treatment of ovarian cancer in the last decade [12]. Recent reviews of the literature have shown that oncological outcomes in this context depend directly on intrinsic tumor factors and do not differ according to the surgical intervention or technique. Reported recurrence rates are 11% for FIGO stage I and up to 29% for FIGO stage II ovarian cancer [28]. The tumor grade has been determined to be as important as the FIGO stage, and some groups recommend that only G1 ovarian tumors be considered eligible for FSS [29,30,31]. In contrast, we identified no significant association of the tumor grade with OS or PFS, but at least similar tendencies in univariate analysis in this study. Bercow et al. stated in their review of the literature on post-FSS outcomes that the use of FSS to treat G3 epithelial ovarian cancer was considered to be controversial in many of 44 included studies, and Vergote et al. described in their retrospective study including 1545 patients with early ovarian cancer that the grade of differentiation was the most powerful prognostic indicator [29,30]. Based on the findings of their retrospective multicenter study, including 34 patients with early ovarian cancer treated with FSS, Morice et al. recommended that the procedure be considered only for stage IA G1 tumors [31]. The lack of effect of the tumor grade on oncological outcomes in the present study may be because 52% of the patients who underwent FSS had G1 tumors. However, the difference in tumor grades between the RS and FSS cohorts was not significant, rebutting the assumption of a missing effect of the grade. Our findings are thus in contrast to those of Fruscio et al., Bercow et al. and Morice et al., who reported that FSS was feasible only for G1/2 tumors [20,29,31]. Further studies of the effect of the tumor grade on oncologic outcomes after laparoscopic FSS are needed. Patients should nevertheless be informed that RS may not necessarily improve their oncological outcomes, as the poorest survival seems to be related to the natural history of the disease and FIGO stage and not to the use of more conservative treatment [32,33].

To our knowledge, this study is one of few to investigate the role of laparoscopic FSS in the treatment of ovarian cancer relative to that of an RS using matched patient cohorts (Table 5). Only one other group has examined the rates of recurrence and death after laparoscopic FSS and deemed FSS to be oncologically safe, but patients were not matched with a comparison group in their study [27]. In that retrospective multicenter study including 48 patients with early-stage ovarian cancer who underwent laparoscopic FSS, the recurrence rate was 15%, and the disease-related death rate was 5% over a median follow-up period of 24 months [27]. We observed a recurrence rate of 28% over a median follow-up period of 36 months and a five-year survival rate of 93%, which is worse than the results reported by Gallotta et al. [27]. One possible explanation for this difference is that Gallotta et al. examined outcomes over a shorter follow-up period and included ten patients with germ cell tumors, potentially leading to a better prognosis. In two retrospective studies including 36 and 307 patients, respectively, with early-stage ovarian cancer, Ditto et al. reported that FSS, performed in 18 and 70 patients, respectively, is as oncologically effective as RS with no increase in the recurrence risk [34,35]. In the first of these studies, 18 patients with FIGO stage I ovarian cancer were matched with 18 patients undergoing RS according to the stage, histological type, and grade, and PFS did not differ between groups, in line with our findings; in the present study, however, we did not match patients according to the histological type or grade, and we cannot comment on the potential impacts of these factors on PFS or OS [34]. In the second study, the tumor stage was the only factor affecting the PFS of 70 patients who underwent FSS and 237 patients who underwent RS on multivariate analysis, in line with our findings [35]. In both studies, however, few (3 and 12, respectively) patients underwent laparoscopic surgery, and the authors did not examine the potential impact of laparoscopic FSS on oncological outcomes [34,35]; in contrast, we examined only patients who underwent laparoscopic surgery. In their review article, Fagotti et al. stated that they found no randomized controlled trial examining laparoscopic FSS but deemed minimally invasive surgery to be safe and adequate based on findings for the use of laparoscopic surgery or FSS in the treatment of early-stage ovarian cancer; they emphasized the advantages of these approaches, especially FSS, due to the likelihood of adhesions, pelvic inflammation, and functional anomalies potentially impairing fertility [13].

FSS has been shown to be an alternative in terms of oncological outcomes. The loss of reproductive capability and surgically induced menopause not only negatively affect the quality of life and survivorship of young women with ovarian cancer but also increase the risks of coronary heart disease, osteoporosis, and cognitive dysfunction in this population [19,36].

In terms of fertility outcomes, the baby take-home rate in the FSS cohort was 75% in this study. Twenty-six percent of patients were pregnant at least once after FSS, in agreement with the reporting of a pregnancy rate of 27.8% [28]. Jiang et al. observed an 80% post-FSS successful pregnancy rate in their retrospective analysis, including 108 patients undergoing FSS or RS; as we did not consider how many women in our cohort tried to conceive, comparison with this rate is not applicable [37]. Bercow et al. indicated that delivery rates ranged from 76% to 96% in the studies included in their systematic review [29]. As the desire to conceive after FSS has been reported to be as low as 25%, we cannot reproduce the delivery rate in our cohort in a statistically reliable manner [38]. The median gestational age at the time of delivery of 38 weeks and the spontaneous delivery rate of 72% in our cohort reflect the “natural” and safe character of pregnancies after FSS, as emphasized by others. In a retrospective analysis including 148 patients who conceived at least three months after surgery for early-stage ovarian cancer, Nitecki et al. observed no increased risk of adverse obstetric outcomes [39]. Alternatives such as ovarian tissue cryopreservation and transplantation have recently become available, but no clinical trial has established their efficacy or safety for women with ovarian cancer, as the risk of tumor cell dissemination is too high to allow for comment on such alternatives in guidelines [28].

A limitation of the present study is its retrospective design, but prospective randomized studies on this topic have not been published. Thus, the present study has contributed to the accumulation of evidence on this clinical question. In addition, given the inhomogeneity of the median age in the FSS and RS groups, an age-related distribution of risk factors could have been missed in our analysis. Women who are counseled on FSS tend to be younger and healthier, with fewer comorbidities, and thus tend to have more favorable oncological outcomes than older women. Younger patients also tend to have more favorable tumor biology; in the present study, 38% of patients who underwent FSS had mucinous ovarian cancer, which may have positively affected survivorship and led to potential bias [33]. Due to the lack of information on the number of women who tried to conceive during the study period and received cytostatic therapy but did not achieve pregnancy, we were not able to calculate the pregnancy rate or evaluate the impact of concomitant oncological therapies in this cohort. Finally, the present study was conducted only in high-volume comprehensive cancer centers with quality-controlled procedural standards, including comprehensive surgical staging, which limits the generalization of the results to other tertiary hospitals. Future studies with case–control matching for FIGO stage, histological subtype, and tumor grading would reduce potential biases and allow the drawing of further conclusions about the oncologic safety of FSS in high-risk collectives (e.g., G3 tumors).

**Table 5:** Summary of the literature on FSS. Fertility-sparing surgery (FSS), Fédération Internationale de Gynécologie et d’Obstétrique (FIGO).

First Author, Year of Publication	Number of Patients Included	FIGO Stage	Surgical Approach	Oncologic Outcomes	Fertility Outcomes
Wright, 2009 [19]	754 (RS) vs. 432 (FSS)	IA, IC	Laparoscopy	FSS no significant effect on survival	-
Fruscio, 2016 [20]	789 (RS) vs. 240 (FSS)	I	Laparotomy	FSS no significant effect on survival	-
Crafton, 2020 [21]	6728 (RS) vs. 2289 (FSS)	I-IV	Not discussed	FSS no significant effect on survival in FIGO stage I	-
Bercow, 2021 [29]	review, 44 studies	I	Not discussed	FSS no significant effect on survival in FIGO stage IA-B, G1–2	Fertility rates from 76% to 96%
Morice, 2005 [31]	0 (RS) vs. 34 (FSS)	I	Laparoscopy and laparotomy	FSS no significant effect on survival in FIGO stage IA, G1	27% pregnancy rate
Gallotta, 2014 [27]	252 (RS) vs. 48 (FSS)	I	Laparoscopy	FSS no significant effect on survival	-
Ditto, 2014 [34]	18 (RS) vs. 18 (FSS)	I	Laparotomy	FSS no significant effect on survival	-
Ditto, 2015 [35]	237 (RS) vs. 70 (FSS)	I	Laparotomy	FSS no significant effect on survival	-
Fagotti, 2016 [13]	review, 8 studies	I	Laparoscopy	FSS no significant effect on survival	-
Jiang, 2017 [37]	56 (RS) vs. 52 (FSS)	I	Laparotomy	FSS no significant effect on survival in FIGO stage I, G1–2	80% pregnancy rate
Nitecki, 2021 [39]	306 (RS) vs. 153 (FSS)	I	Not discussed	-	no increased risk of adverse obstetric outcomes

## 5. Conclusions

FSS seems to be oncologically safe and applicable in the treatment of early-stage ovarian cancer, but further studies are needed to evaluate its application for ovarian cancer of more advanced FIGO stages. Fertility outcomes of FSS were promising in this multicentric analysis. The decision to pursue FSS should be individualized based on patient–provider counseling, disease characteristics, and tumor stage and biology to achieve maximum oncological safety.

## Figures and Tables

**Table 1:** Patient and tumor characteristics. Radical surgery (RS), fertility-sparing surgery (FSS), Fédération Internationale de Gynécologie et d’ Obstétrique (FIGO), progression-free survival (PFS), overall survival (OS).

Variable	RS *n* = 40	FSS *n* = 40	*p*
**Age (years; median, range)**	56 (29–81)	28 (15–54)	**≤0.01**
**FIGO stage**			1.0
Ia	23 (57%)	23 (57%)	
Ib	0 (0%)	0 (0%)	
Ic	15 (38%)	15 (38%)	
IcI	10 (25%)	10 (25%)	
IcII	2 (5%)	2 (5%)	
IcIII	3 (8%)	3 (8%)	
II	2 (5%)	2 (5%)	
**Subtype**			**≤0.01**
Serous	22 (55%)	13 (32%)	
Endometrioid	10 (25%)	5 (12%)	
Clear cell	3 (8%)	2 (5%)	
Mucinous	2 (5%)	15 (38%)	
Seromucinous	2 (5%)	2 (5%)	
Mixed	1 (2%)	3 (8%)	
**Grading**			0.07
G1	11 (28%)	21 (52%)	
G2	16 (40%)	11 (28%)	
G3	13 (32%)	8 (20%)	
**Resection**			1.0
R0	40 (100%)	40 (100%)	
R1	0 (0%)	0 (0%)	
**Adjuvant chemotherapy**			0.25
yes	17 (43%)	12 (30%)	
no	23 (57%)	28 (70%)	
**Recurrence**			0.80
yes	9 (23%)	11 (28%)	
no	31 (77%)	29 (72%)	
**Intraoperative complications**			0.31
yes	1 (2%)	0 (0%)	
no	39 (98%)	40 (100%)	
**Organ injury**			
Intestine			0.31
yes	1 (2%)	0 (0%)	
no	39 (98%)	40 (100%)	
Bladder			1.0
yes	0 (0%)	0 (0%)	
no	40 (100%)	40 (100%)	
Ureter			1.0
yes	0 (0%)	0 (0%)	
no	40 (100%)	40 (100%)	
Vessels			1.0
yes	0 (0%)	0 (0%)	
no	40 (100%)	40 (100%)	
Other			1.0
yes	0 (0%)	0 (0%)	
no	40 (100%)	40 (100%)	
**Bleeding**			1.0
yes	0 (0%)	0 (0%)	
no	40 (100%)	40 (100%)	
**Resuscitation**			1.0
yes	0 (0%)	0 (0%)	
no	40 (100%)	40 (100%)	
**Skin emphysema**			1.0
yes	0 (0%)	0 (0%)	
no	40 (100%)	40 (100%)	
**Postoperative complications**			0.46
yes	5 (12%)	3 (7%)	
no	35 (88%)	37 (93%)	
**Clavien Dindo**			
Grade I	0 (0%)	1 (2%)	0.31
Grade II	1 (2%)	0 (0%)	0.31
Grade III	3 (7%)	2 (5%)	0.64
Grade IV	1 (2%)	0 (0%)	0.31
Grade V	0 (0%)	0 (0%)	1.0
**Postoperative length of hospital stay (days; median, range)**	9.5 (0–27)	4 (0–12)	**0.04**
**Follow-up (months; median, range)**	54 (1–275)	36 (3–150)	0.69
**PFS (months; median, range)**	150 (5–150)	150 (3–150)	0.61
**OS (months; median, range)**	50 (1–275)	36 (3–150)	0.65
**3-year PFS**	85% (SE 0.06)	80% (SE 0.06)	0.78
**5-year PFS**	72% (SE 0.08)	68% (SE 0.09)	0.78
**3-year OS**	90% (SE 0.06)	93% (SE 0.04)	0.61
**5-year OS**	90% (SE 0.06)	93% (SE 0.04)	0.61

**Table 2:** Univariate analysis for the risk of recurrence. Fédération Internationale de Gynécologie et d’Obstétrique (FIGO).

Variable	OR	95% CI	*p*
**Age**	0.99	0.97–1.03	0.81
**Surgical approach**			
Radical surgery vs. Fertility-sparing surgery	0.76	0.27–2.12	0.60
**FIGO stage**			
I vs. II	6.11	0.52–71.44	0.15
**Grading**			
G1 vs. G3	0.84	0.44–1.61	0.59
**Subtype**			
Serous vs. endometrioid	1.05	0.26–4.24	0.95
Serous vs. clear cell	0.72	0.07–7.42	0.78
Serous vs. mucinous	0.89	0.22–3.52	0.86
Serous vs. seromucinous	0.96	0.09–10.58	0.97
Serous vs. mixed	2.88	0.35–23.92	0.33

**Table 3:** Univariate analysis for the risk of death. Fédération Internationale de Gynécologie et d’Obstétrique (FIGO).

Variable	OR	95% CI	*p*
**Age**	1.01	0.97–1.06	0.57
**Surgical approach**			
Radical surgery vs. Fertility-sparing surgery	1.06	0.20–5.61	0.95
**FIGO stage**			
I vs. II	0	0–1	0.99
**Grading**			
G1 vs. G3	2.17	0.73–6.42	0.16
**Subtype**			
Serous vs. endometrioid	0	0–1	0.99
Serous vs. clear cell	0	0–1	0.99
Serous vs. mucinous	0.36	0.04–3.38	0.37
Serous vs. seromucinous	0	0–1	0.99
Serous vs. mixed	0	0–1	0.99

**Table 4:** Pregnancy Outcomes in patients treated with fertility-sparing surgery for early ovarian cancer. In vitro fertilization (IVF).

Variable	*n* (%)
Fertility follow-up exists	31/40 (77.5%)
Time from surgery to pregnancy (months; median, range)	12 (6–37)
Chemotherapy prior to conception	2 (25%)
One or more pregnancies	8 (26%)
Conception with IVF	3 (38%)
Spontaneous conception	5 (63%)
Full-term pregnancies	7 (23%)
Miscarriages	2 (6%)
**Medically assisted reproduction**	
IVF without pregnancy	1 (3%)
Cryoconservation	2 (6%)
Gestational age at time of delivery (weeks; median, range)	38 (33–40)
**Mode of delivery**	
Spontaneous delivery	5 (72%)
Elective C-section	1 (14%)
Secondary C-section	1 (14%)
Completion surgery after pregnancy	3 (8%)
Completion surgery without pregnancy	4 (10%)
Live birth rate	225 (for every 1000 people)
Baby take-home rate	75% (3/4)

## Data Availability

The dataset used and analyzed during the current study is available from the corresponding author upon reasonable request.

## References

[1] Jayson G.C., Kohn E.C., Kitchener H.C., Ledermann J.A. (2014). Ovarian cancer. Lancet.

[2] Siegel R.L., Miller K.D., Jemal A. (2016). Cancer statistics. CA-Cancer J. Clin..

[3] Benedet J.L., Bender H., Jones H., Ngan H.Y., Pecorelli S. (2000). FIGO staging classifications and clinical practice guidelines in the management of gynecologic cancers. FIGO Committee on Gynecologic Oncology. Int. J. Gynaecol. Obstet..

[4] Heintz A.P., Odicino F., Maisonneuve P., Quinn M.A., Benedet J.L., Creasman W.T., Ngan H.Y., Pecorelli S., Beller U. (2006). Carcinoma of the ovary. FIGO 26th Annual Report on the Results of Treatment in Gynecological Cancer. Int. J. Gynaecol. Obstet..

[5] Sant M., Allemani C., Santaquilani M., Knijn A., Marchesi F., Capocaccia R. (2009). EUROCARE-4. Survival of cancer patients diagnosed in 1995-1999. Results and commentary. Eur. J. Cancer.

[6] Siegel R., Naishadham D., Jemal A. (2012). Cancer statistics, 2012. CA-Cancer J. Clin..

[7] National Cancer Institute Cancer Stat. Facts: Ovarian Cancer. https://seer.cancer.gov/statfacts/html/ovary.html.

[8] Mathews T.J., Hamilton B.E. (2016). Mean Age of Mothers is on the Rise: United States, 2000–2014. NCHS Data Brief.

[9] Carter J., Chi D.S., Brown C.L., Abu-Rustum N.R., Sonoda Y., Aghajanian C., Levine D.A., Baser R.E., Raviv L., Barakat R.R. (2010). Cancer-related infertility in survivorship. Int. J. Gynecol. Cancer.

[10] Carter J., Rowland K., Chi D., Brown C., Abu-Rustum N., Castiel M., Barakat R. (2005). Gynecologic cancer treatment and the impact of cancer-related infertility. Gynecol. Oncol..

[11] Lee S.J., Schover L.R., Partridge A.H., Patrizio P., Wallace W.H., Hagerty K., Beck L.N., Brennan L.V., Oktay K., American Society of Clinical Oncology (2006). American Society of Clinical Oncology recommendations on fertility preservation in cancer patients. J. Clin. Oncol..

[12] Medeiros L.R., Rosa D.D., Bozzetti M.C., Rosa M.I., Edelweiss M.I., Stein A.T., Zelmanowicz A., Ethur A.B., Zanini R.R. (2008). Laparoscopy versus laparotomy for FIGO Stage I ovarian cancer. Cochrane Database Syst. Rev..

[13] Fagotti A., Perelli F., Pedone L., Scambia G. (2016). Current Recommendations for Minimally Invasive Surgical Staging in Ovarian Cancer. Curr. Treat. Options Oncol..

[14] Satoh T., Hatae M., Watanabe Y., Yaegashi N., Ishiko O., Kodama S., Yamaguchi S., Ochiai K., Takano M., Yokota H. (2010). Outcomes of fertility-sparing surgery for stage I epithelial ovarian cancer: A proposal for patient selection. J. Clin. Oncol..

[15] Gershenson D.M. (2012). Treatment of ovarian cancer in young women. Clin. Obstet. Gynecol..

[16] National Comprehensive Cancer Network NCCN Clinical Practice Guidelines in Oncology. Ovarian Cancer. https://www.nccn.org/professionals/physician_gls/default.aspx#site.

[17] Dindo D., Demartines N., Clavien P.A. (2004). Classification of surgical complications: A new proposal with evaluation in a cohort of 6336 patients and results of a survey. Ann. Surg..

[18] Deutsche Krebsgesellschaft, Deutsche Krebshilfe, AWMF Leitlinienprogramm Onkologie S3-Leitlinie Diagnostik, Therapie und Nachsorge maligner Ovarialtumoren, Langversion 5.0—September 2021. https://www.leitlinienprogramm-onkologie.de/fileadmin/user_upload/Downloads/Leitlinien/Ovarialkarzinom/Version_5/LL_Ovarialkarzinom_Langversion_5.0.pdf.

[19] Wright J.D., Shah M., Mathew L., Burke W.M., Culhane J., Goldman N., Schiff P.B., Herzog T.J. (2009). Fertility preservation in young women with epithelial ovarian cancer. Cancer.

[20] Fruscio R., Ceppi L., Corso S., Galli F., Dell’Anna T., Dell’Orto F., Giuliani D., Garbi A., Chiari S., Mangioni C. (2016). Long-term results of fertility-sparing treatment compared with standard radical surgery for early-stage epithelial ovarian cancer. Br. J. Cancer.

[21] Crafton S.M., Cohn D.E., Llamocca E.N., Louden E., Rhoades J., Felix A.S. (2020). Fertility-sparing surgery and survival among reproductive-age women with epithelial ovarian cancer in 2 cancer registries. Cancer.

[22] Vergote I. (2004). Role of surgery in ovarian cancer: An update. Acta Chir. Belg..

[23] Muzii L., Angioli R., Zullo M., Panici P.B. (2005). The unexpected ovarian malignancy found during operative laparoscopy: Incidence, management, and implications for prognosis. J. Minim. Invasive Gynecol..

[24] Schorge J.O., Eisenhauer E.E., Chi D.S. (2012). Current surgical management of ovarian cancer. Hematol./Oncol. Clin. N. Am..

[25] Zimmermann J.S.M., Radosa J.C., Radosa M.P., Sklavounos P., Schweitzer P.A., Solomayer E.F. (2021). Survey of current practices and opinions of German Society of Gynecologic Endoscopy members regarding the treatment of ovarian neoplasia by robotic surgery. Arch. Gynecol. Obstet..

[26] Ghezzi F., Cromi A., Siesto G., Serati M., Zaffaroni E., Bolis P. (2009). Laparoscopy staging of early ovarian cancer. Int. J. Gynecol. Cancer.

[27] Gallotta V., Ghezzi F., Vizza E., Chiantera V., Ceccaroni M., Franchi M., Fagotti A., Ercoli A., Fanfani F., Parino C. (2014). Laparoscopic staging of apparent early stage ovarian cancer: Results of a large, retrospective, multi-institutional series. Gynecol. Oncol..

[28] du Bois A., Heitz F., Harter P. (2013). Fertility-sparing surgery in ovarian cancer: A systematic review. Onkologie.

[29] Bercow A., Nitecki R., Brady P.C., Rauh-Hain J.A. (2021). Outcomes after Fertility-sparing surgery for women with ovarian cancer: A systematic review of the literature. J. Minim. Invasive Gynecol..

[30] Vergote I., De Brabanter J., Fyles A., Bertelsen K., Einhorn N., Sevelda P., Gore M.E., Kaern J., Verrelst H., Sjövall K. (2001). Prognostic importance of degree of differentiation and cyst rupture in stage I invasive epithelial ovarian carcinoma. Lancet.

[31] Morice P., Leblanc E., Rey A., Baron M., Querleu D., Blanchot J., Duvillard P., Lhommé C., Castaigne D., Classe J.M. (2005). Conservative treatment in epithelial ovarian cancer: Results of a multicentre study of the GCCLCC (Groupe des Chirurgiens de Centre de Lutte Contre le Cancer) and SFOG (Société Francaise d’Oncologie Gynécologique). Hum. Reprod..

[32] Kommoss S., Harter P., Traut A., Strutas D., Riegler N., Buhrmann C., Gomez R., du Bois A. (2009). Compliance to consensus recommendations, surgeon’s experience, and introduction of a quality assurance and management program: Influence on therapy of early-stage ovarian carcinoma. Int. J. Gynecol. Cancer.

[33] Salem W.H., Letourneau J.M., Chan J., Chan S.W., Cedars M., Rosen M.P. (2017). Cancer survivors of gynecologic malignancies are at risk for decreased opportunity for fertility preservation. Contracept. Reprod. Med..

[34] Ditto A., Martinelli F., Lorusso D., Haeusler E., Carcangiu M., Raspagliesi F. (2014). Fertility sparing surgery in early stage epithelial ovarian cancer. J. Gynecol. Oncol..

[35] Ditto A., Martinelli F., Bogani G., Lorusso D., Carcangiu M., Chiappa V., Reato C., Donfrancesco C., De Carrillo K.J., Raspagliesi F. (2015). Long-term safety of fertility sparing surgery in early stage ovarian cancer: Comparison to standard radical surgical procedures. Gynecol. Oncol..

[36] Oktay K., Harvey B.E., Partridge A.H., Quinn G.P., Reinecke J., Taylor H.S., Wallace W.H., Wang E.T., Loren A.W. (2018). Fertility Preservation in Patients with Cancer: ASCO Clinical Practice Guideline Update. J. Clin. Oncol..

[37] Jiang X., Yang J., Yu M., Xie W., Cao D., Wu M., Pan L., Huang H., You Y., Shen K. (2017). Oncofertility in patients with stage I epithelial ovarian cancer: Fertility-sparing surgery in young women of reproductive age. World J. Surg. Oncol..

[38] Ghezzi F., Cromi A., Fanfani F., Malzoni M., Ditto A., De Iaco P., Uccella S., Gallotta V., Raspagliesi F., Scambia G. (2016). Laparoscopic fertility-sparing surgery for early ovarian epithelial cancer: A multi-institutional experience. Gynecol. Oncol..

[39] Nitecki R., Clapp M.A., Fu S., Lamiman K., Melamed A., Brady P.C., Kaimal A., Del Carmen M.G., Woodard T.L., Meyer L.A. (2021). Outcomes of the First Pregnancy After Fertility-Sparing Surgery for Early-Stage Ovarian Cancer. Obstet. Gynecol..

